# Evaluation of Diagnostic Imaging Capacity and the Role for Point-of-Care Ultrasound (POCUS) within the Zanzibar Health System

**DOI:** 10.24908/pocus.v6i1.14763

**Published:** 2021-04-22

**Authors:** Abiola A Fasina, Anthony J Dean, Nova L Panebianco, Frances S Shofer, Omar Ali, Mwajuma Yahya, Salim Ismail, Patricia C Henwood

**Affiliations:** 1 Emergency Healthcare Consultants Lagos Nigeria; 2 Point-of-care Ultrasound in Resource-limited Environments Philadelphia, PA USA; 3 Department of Emergency Medicine, University of Pennsylvania Hospital Philadelphia, PA USA; 4 Zanzibar Diaspora Association; 5 Department of Radiology, Mnazi Moja Hospital Unguja Zanzibar; 6 Department of Emergency Medicine, Thomas Jefferson University Hospital Philadelphia, PA USA

**Keywords:** Zanzibar, ultrasound, point of care, obstetrics, training, Tanzania, healthcare, imaging

## Background

Continually, diagnostic imaging is neglected when considering strategies for quality improvement in healthcare delivery in resource-limited settings. The World Health Organization has advocated for expanded global access to ultrasound teaching and technology for health providers 1, 2, as two-thirds of the world’s population currently has no access to imaging technologies. Supporting CT or MRI can be particularly challenging due to the cost of the initial equipment investment combined with infrastructure issues including maintenance and repair requirements, and training of technologists, thus the call for scale up of diagnostic ultrasound and x-ray capacity in particular in rural healthcare services 3. 

Point-of-care ultrasound (POCUS) has been employed in resource-limited settings has demonstrated improvements in clinical decision-making and often leads to an immediate change in management 4, 5. Purchase and maintenance costs of ultrasound machines are relatively inexpensive 5, 6, and are becoming more portable and affordable 7. POCUS does not depend on specialized technologists or radiologists, both of which can be rare in resource-limited clinical settings 1. There are savings of time and personnel engendered by the need for only one provider to acquire, interpret, and act on the test, and there may be direct cost savings associated 8.

Zanzibar is an archipelago consisting of two main islands which functions as a semi-autonomous region of the United Republic of Tanzania, with its own ministry of health. The Zanzibar Ministry of Health completed a preceding health system assessment and identified imaging as a major gap. Due to the very limited baseline radiology capacity (one radiologist in Zanzibar), they requested input of this research team to further evaluate the current role of ultrasound and suggest development of a POCUS training program thereafter. 

There is an overall shortage of skilled clinicians in Zanzibar 9. At the time of this study, there was one radiologist in Zanzibar for a population over one million. Subsequently, two radiologists have joined the staff at Mnazi Moja hospital bringing the total number of radiologists on the island to three. While there is access to public health facilities, the range of health care services provided is limited, including diagnostic imaging. At the same time, Zanzibar demographics reflect a young age structure with high birth, maternal mortality, and overall death rates, as well as low rates of contraceptive usage 10, 11. The World Health Organization (WHO) estimates only half of Tanzanian women have regular antenatal care and deliver with a skilled birth attendant present 12, and overall access to comprehensive reproductive healthcare is limited 11. Prior research shows one of the greatest impacts of ultrasound incorporated into clinical care is in obstetric conditions 6, 13, 14, 15. POCUS has also proved useful in trauma, HIV/TB, pulmonary, cardiac, gynecology, hepatobiliary disease, genitourinary disease, mass casualty events, and tropical/infectious diseases 16, 17, 18, 19, 20. 

The primary objective of this study was to evaluate diagnostic imaging capacity within the Zanzibar health system, with a focus on ultrasound availability, practitioners’ current use of ultrasound, knowledge of and interest in ultrasound use. The data collected from this assessment was collated into a needs assessment report to the Ministry of Health, and served as the background to a POCUS training program initiated in conjunction with the Zanzibar Ministry of Health. 

## Methods

**Figure 1:**
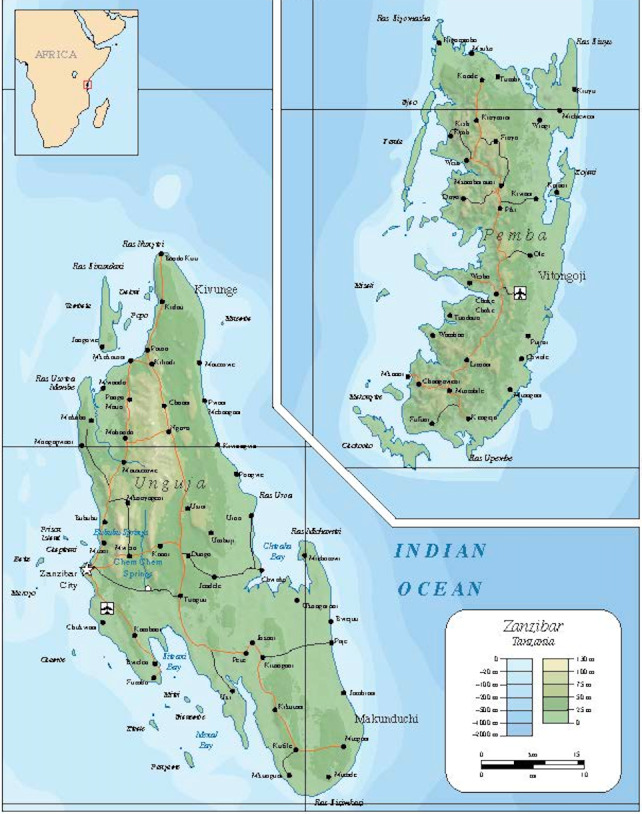
Map of Zanzibar’s 2 Islands. This image ("Map_of_Zanzibar_Archipelago-en.svg" by Oona Räisänen) is reproduced under a CC BY-SA 4.0 license through Wikipedia Commons .

This is a mixed methodology cross sectional design using both a quantitative survey and focused personal interviews (FPIs). The needs assessment occurred between July and August of 2015 at eight public hospitals across Zanzibar. Diagnostic imaging capacity with a focus on ultrasound was evaluated at five rural and district hospitals on Pemba, two district hospitals on Unguja and also at Mnazi Mmoja Hospital (MMH), the referral hospital for Zanzibar (Figure 1). One day was spent at each site meeting clinical and administrative members of the hospital staff as well as imaging technicians. Where needed, follow up visits were made to meet previously unavailable respondents.

The protocol was deemed exempt by the Institutional Review Board at the University of Pennsylvania and approved by the Ministry of Health of Zanzibar. This was deemed exempt due to the focus on health system capacity assessment, limited risk to clinician participants in completing de-identified surveys, and no patient involvement in this study. Respondents were informed of the study objectives prior to completing the survey and provided verbal consent prior to participation. Survey and interview information collected from participants were done in a de-identified fashion, and participants did not receive any incentives.

This was a convenience sample of healthcare workers on duty at the time the investigator was at each site. Convenience sampling was utilized because apart from Mnazi Moja, these hospitals have a small number of clinical staff and it was possible to meet the majority of them within one to two visits. Targeted individuals included medical officers, assistant medical officers, physicians, nurses, midwives and imaging technicians in plain film radiography and ultrasound. After completing a self-administered quantitative ultrasound needs-assessment survey, respondents also completed a focused personal interview (FPI) with the principal investigator. The needs assessment tool was modeled on one previously published 21, used for similar purposes in Colombia 22. It was modified to include items related to practice around management and transfer of patients to referral hospitals. The questionnaire was administered in English (the language of instruction at Zanzibar schools). 

In addition to basic demographics, the survey tool included items in the following domains related to ultrasound: current practice patterns, integration into workflow, extent of prior exposure, desire for training and barriers to use. The questionnaire has 26 items grouped into themes and requires approximately fifteen minutes to complete 21, 22.The first section focused on details of the individual practitioner’s practice location such as availability of specialist care and ultrasound as well as referral hospital transfer patterns. The second section focused on individual practice. The last section collected information regarding familiarity with ultrasound including any prior formal training. 

Focused personal interviews (FPIs) were conducted by the principal investigator. At each site, a senior clinician or imaging technician, if available, was asked about all current imaging equipment and staff to create a detailed inventory of existing ultrasound machines, sonographers and radiologists. Imaging capacity for other modalities such as plain film radiography, computed tomography (CT), and MRI were also investigated. Information about training, most common applications of ultrasounds, methods of procurement of ultrasound equipment including gel and supplies, patient payment schemes for ultrasound scans and current capacity to manage acute conditions were assessed. Sessions occurred in English though an accompanying local coordinator, present during all interviews, occasionally assisted with interpreting difficult concepts in Swahili. On average, these sessions lasted fifteen minutes and were conducted in a private location convenient to the respondent to encourage open discussion. 

The FPI administrator kept careful thematic notes during the sessions to capture qualitative data necessary to provide a programmatic context to the surveys. Qualitative data was analyzed using a simplified grounded theory approach in which specific themes were identified. Releavant thematic patterns emerged after analysis of the data collected to inform the results. The interview process was then finetuned over multiple iterations using a comparative method. Summary statistics such as frequencies and percentages were used to describe the survey data. 

## Results

At the time of the study, there were nine total ultrasound machines present at six of the eight public hospitals assessed. All had x-ray, but only Mnazi Mmoja had a CT scanner at the time of the study. At the time of the study, there was no MRI capacity in Zanzibar and twelve sonographers and one radiologist served all of the public hospitals in Zanzibar. Since then, Mnazi Moja hospital has acquired an MRI machine which is the only one available in Zanzibar. A private facility now also has a CT scanner. Two radiologists have joined the staff at Mnaza Moja Hospital bringing the total number of radiologists in Zanzibar up to three. A total of 40 ultrasound-focused surveys were collected, 37 in person and 3 electronically. All respondents worked at public hospitals across the two main islands of Zanzibar and were drawn from the full spectrum of healthcare workers at various stages professionally. Fifty-four percent of respondents were female and 32.5% were physicians (Table 1). 

**Table 1:** Study Population (N=40*)

Demographic		N	(%)
Gender	Female	21	(53.8)
Level of Training	Physician	13	(32.5)
	Assistant Medical Officer	7	(17.5)
	Midwife	5	(12.5)
	Imaging Technician (XR or Ultrasound)	4	(10.0)
	Clinical Officer	4	(10.0)
	Clinical Health Nurse	3	(7.5)
	Medical Student	2	(5.0)
	Non-respondents	2	(5.0)
Medical Specialty	General Medicine	12	(38.7)
	Obstetrics and Gynecology	11	(38.7)
	General Surgery	4	(12.9)
	Pediatrics	1	(3.2)
	Ophthalmology	1	(3.2)
	Psychiatry	1	(3.2)
Practice Location	Rural	12	(30.0)
	District	7	(17.5)
	Referral	21	(52.5)
Reported Years of Clinical Experience	< 1 year	7	(17.5)
	1 to 5 years	16	(40.0)
	5 to 10 years	4	(10.0)
	> 10 years	13	(32.5)
Duration of Practice at Current Location	0 to 6 months	3	(7.5)
	6 to 12 months	7	(17.5)
	1 to 2 years	11	(27.5)
	2 to 4 years	7	(17.5)
	>5 years	12	(30.0)
Some formal ultrasound training		3	(7.7)
Prior Ultrasound Experience		4	(10.0)

There was limited prior experience with ultrasound among the healthcare workers surveyed. Only four (10%) respondents had prior formal ultrasound training and prior ultrasound experience. All respondents indicated interest in learning POCUS with 71.8% indicating a ‘high’ level of interest. Seventy percent of respondents expressed an interest in learning obstetric ultrasound. The second and third highest ranked modalities of interest were liver (22.5%) and trauma (17.5%) ultrasound. Respondents also indicated that when available, US is the initial imaging modality used for abdominal complaints. Twelve (30%) of respondents stated they would be willing to spend more than 10 hours weekly learning POCUS, while 19 (47.5%) respondents indicated they would be willing to spend 4 – 10 hours weekly. Five respondents (12.5%) indicated they would be available for less than 4 hours weekly. The remaining four respondents (10%) were split equally into two groups: two (5%) stated they had no time and two (5%) did not respond. 

Respondents also indicated most common barriers to ultrasound use were a lack of teachers (40%), a lack of machines (28%), and no financial incentive (21%). HCW turnover was noted to be relatively low in this study; thirty-three (82.5%) of respondents had spent more than one year at their current practice location and 13 (32.5%) had spent more than five years at their current practice location. Of the thirty-six respondents who were clinicians, thirty-one (86.1%) reported that they were comfortable performing vaginal deliveries independently and sixteen (44.4%) reported that they were comfortable performing C-sections independently. 

Sixteen respondents agreed to a focused personal interview. The most important themes that emerged from the FPIs were high demand for ultrasound services and limited ability to meet the need. Results from the survey administered indicated that 75% of HCPs had access to plain radiography and 80% to technologist-performed ultrasound. FPIs revealed persistent perceived difficulties with imaging services. The major themes from the FPIs were poor access to functioning machines and limited ability to maintain machines. The technicians and sonographers within the sample reported using old machines that break down frequently and a lack of access to replacement parts or skilled engineers to service existing machines. Radiology staff reported that there was high demand for ultrasound services without the necessary resources to meet the need in terms of available sonographers and machines. Lastly, the interviews revealed that delays within the system makes the procurement of gel and supplies as all such requests are processed through a central office at the MOH resulting in shortage of supplies. Respondents indicated that a patient cost-sharing scheme was started to mitigate this effect. With the patient cost-sharing scheme, every patient who benefitted from diagnostic ultrasound paid a small fee into a common pool of funds to maintain supplies such as ultrasound gel and gloves. Funds generated through this avenue are used to purchase supplies. Despite this, technicians still reported difficulty in maintaining ultrasound supplies. 

HCPs reported poor access and delays particularly at night and on weekends. HCPs on Pemba reported less access at all times due to their distance from Mnazi Mmoja Hospital and the

requirement for boat or flight transport due to their separate island location. Plain film radiography was available at all hospitals but two of the five hospitals on Pemba, (Micheweni and Vitongoji), had no ultrasound capability (see Appendix 2). 

## Discussion

This first detailed evaluation of diagnostic imaging capacity with a focus on the role of ultrasound within the Zanzibar public health demonstrates it to be extremely limited. Only plain films are regularly available outside of the referral hospital, and there was only one radiologist working in the public sector for a population of over one million, at the time of the study. Although ultrasound devices were available at some of the hospitals we evaluated, this study revealed overall limited prior experience and training with ultrasound among clinicians. Though there is a significant degree of interest in education, particularly obstetric applications, the study revealed limited opportunity to acquire formal training, as well as lack of teachers, machines, and financial incentive as barriers to use. 

The World Health organization notes that 80-90% of all diagnostic problems could be solved by basic radiography (x-ray) and ultrasound (US) examinations 23. POCUS has been shown to change clinical decision-making in resource-limited settings, and some of the greatest utility appears to be in trauma and obstetrics 6, 24, 25. The initial interest of this cohort in the obstetric and trauma ultrasound is consistent with the pattern seen in other studies in similar resource-limited settings, and may reflect greater familiarity with the use of ultrasound for these indications 14, 26, 27. 

A study in Liberia showed the introduction of POCUS resulted in changes of management in 62% of cases seen at the tertiary teaching hospital in Monrovia 6. The greatest impact was in cases of pregnancy, echocardiography and focused assessment of sonography in trauma (FAST).^6^ Similar results were reported from Tanzania, Rwanda, and Ghana.^28^ Though a primary focus on obstetrics is of interest in this setting, trauma and abdominal POCUS applications, is of interest to this cohort and may prove useful given the scarcity of CT in this environment. 

Several important barriers to ultrasound use were identified using this survey. The first was a lack of machines. With the exception of Mnazi Moja Hospital (MMH), ultrasound machines were frequently older and donated from abroad. Many were broken down or needed extensive service that was no longer possible due to their age or lack of warranty. Part of creating a sustainable program in POCUS in Zanzibar would involve acquiring durable ultrasound machines with a service plan for repair. Machine access for regular use is necessary to maintain and improve ultrasound skills and create a sustainable program. 

The second identified barrier was the lack of teachers. Developing a sustainable ultrasound training program starts with longitudinal educator engagement, giving trainees the opportunity to practice under careful supervision.^21^, ^29^ Then investing in POCUS trainees that become educators that longitudinally work toward capacity building with their colleagues and communities is ideal. A POCUS training program on Pemba Island in Zanzibar that utilizes a ‘train the trainers’ model garnered considerable success.^30^ Therein, establishing a stable cohort of local POCUS leaders/trainers is integral to achieve self-sustaining integration into a care setting. 

Information from the FPIs revealed that while x-ray and ultrasound are present at most facilities, access to such equipment is poor. HCPs reported difficulty obtaining scans and sonographers reported prolonged turnaround times for ordered scans due to malfunctioning machines and inadequate staff to meet demand as the sonographers who perform the scans also interpret and report on them. This suggests that if POCUS training was provided, efficiency may be increased by clinicians performing and interpreting their own studies, within the relevant clinical context, in real-time.

This study was the initial assessment of ultrasound needs and capabilities within the Zanzibar health system. Data from this study was used to design a training program for HCPs in Zanzibar. Subsequently in 2016, fifteen HCPs from three hospitals on Pemba enrolled in a six-month training program with a special focus on obstetrics. Results from the training program with respect to training modalities, ultrasound skills acquisition and retention are published in a separate article. 

## Limitations

This study assessed a small convenience sample of HCPs encountered in public hospitals across Zanzibar. While every effort was made to interview and survey as many practitioners as possible, some sampling bias may exist and these practitioners may not be representative of the population of HCPs in Zanzibar or their practice patterns. This may also not be representative of resources and HCPs in private settings. This study was led by investigators from outside Zanzibar at the request of the Ministry of Health due to limited radiology capacity in Zanzibar so investigators’ lack of direct experience working in the healthcare system may be an additional limitation. 

At the time of this needs assessment, CT was only available at Mnazi Moja hospital within the public sector and no public hospitals had MRI capability. Although English is the language of instruction in Zanzibar, HCPs have varying levels of comfort with the language. To minimize the effect of language barrier, an interpreter fluent in the native language was present during the FPI interviews. While effort was taken to make respondents feel that their survey answers and FPI discussions were confidential, it is possible responses were tempered or influenced by employment concerns. The scope of this investigation was limited to the survey questionnaire and FPIs. Both tools captured information related to self-reported use of ultrasound and not direct measurements of use or patient outcomes. Finally, this cross-sectional survey does not allow for an assessment of changing patterns of POCUS use over time. 

## Conclusions

Healthcare providers in Zanzibar have limited access to diagnostic imaging and express a high level of interest in learning and integrating point-of-care ultrasound. The lack of teachers and equipment were noted as main barriers to use. Equipment and educational support for a POCUS program could improve care by increasing access to diagnostic imaging. 

## Competing interests

The authors have no relevant financial or non-financial competing interests.
